# Defining the Most Potent Osteoinductive Culture Conditions for MC3T3-E1 Cells Reveals No Implication of Oxidative Stress or Energy Metabolism

**DOI:** 10.3390/ijms25084180

**Published:** 2024-04-10

**Authors:** Alexandra Semicheva, Ufuk Ersoy, Aphrodite Vasilaki, Ioanna Myrtziou, Ioannis Kanakis

**Affiliations:** 1 Chester Medical School, Faculty of Health, Medicine and Society, University of Chester, Chester CH1 4BJ, UK; a.semicheva@chester.ac.uk (A.S.); i.myrtzioukanaki@chester.ac.uk (I.M.); 2 Department of Musculoskeletal & Ageing Science, Institute of Life Course & Medical Sciences (ILCaMS), University of Liverpool, Liverpool L7 8TX, UK; ersoyuf@liverpool.ac.uk (U.E.); vasilaki@liverpool.ac.uk (A.V.)

**Keywords:** MC3T3-E1 subclone 4, mineralisation, oxidative stress, energy metabolism

## Abstract

The MC3T3-E1 preosteoblastic cell line is widely utilised as a reliable in vitro system to assess bone formation. However, the experimental growth conditions for these cells hugely diverge, and, particularly, the osteogenic medium (OSM)’s composition varies in research studies. Therefore, we aimed to define the ideal culture conditions for MC3T3-E1 subclone 4 cells with regard to their mineralization capacity and explore if oxidative stress or the cellular metabolism processes are implicated. Cells were treated with nine different combinations of long-lasting ascorbate (Asc) and β-glycerophosphate (βGP), and osteogenesis/calcification was evaluated at three different time-points by qPCR, Western blotting, and bone nodule staining. Key molecules of the oxidative and metabolic pathways were also assessed. It was found that sufficient mineral deposition was achieved only in the 150 μg.mL^−1^/2 mM Asc/βGP combination on day 21 in OSM, and this was supported by *Runx2*, *Alpl*, *Bglap*, and *Col1a1* expression level increases. NOX2 and SOD2 as well as PGC1α and Tfam were also monitored as indicators of redox and metabolic processes, respectively, where no differences were observed. Elevation in OCN protein levels and ALP activity showed that mineralisation comes as a result of these differences. This work defines the most appropriate culture conditions for MC3T3-E1 cells and could be used by other research laboratories in this field.

## 1. Introduction

Osteoblasts are specialised bone-forming cells that play a crucial role in bone development, homeostasis, and repair [1,2]. The MC3T3-E1 preosteoblastic cell line, derived from mouse calvaria, is widely used as a model system for studying bone formation in vitro, as these cells can differentiate into mature osteoblasts and produce calcified bone matrix [3,4]. Several subclones of MC3T3-E1 cells have been established, each with its unique characteristics and responsiveness to differentiation stimuli [5]. During osteogenic differentiation, the addition of specific supplements, such as ascorbic acid (Asc) and beta-glycerophosphate (βGP), is necessary to induce the osteoblastic phenotype with mineralisation capacity [6].

Ascorbic acid, a vitamin C derivative, is a critical component in the osteogenic differentiation of MC3T3-E1 cells. It serves as a cofactor for prolyl hydroxylase, an enzyme involved in collagen synthesis, which is essential for bone matrix formation [7]. Ascorbic acid also has antioxidant properties that protect against oxidative stress, which can impair osteoblast function [8]. On the other hand, βGP, an organic phosphate source, is another crucial component in hydroxyapatite deposition on bone matrix produced by MC3T3-E1 cells. It serves as a substrate for alkaline phosphatase, an enzyme which plays a pivotal role in mineralisation by hydrolysing phosphate esters [9,10]. The supplementation of βGP enhances the osteogenic commitment of MC3T3-E1 cells. Researchers have explored the effects of different concentrations of βGP on osteoblast behaviour and found that an optimal range (2–10 mM) promotes mineralisation [11,12,13]. However, it has been reported that concentrations above 5 mM result in non-specific staining of the bone nodules being formed by mouse osteoblasts [6,14].

In the current literature, there is an inconsistency between different in vitro experiments about the use of specific concentrations for Asc and βGP and a lack of understanding of how essential mechanisms in osteoblast differentiation and mineralisation, like oxidative stress and the energy metabolism, are affected by their concentration. Oxidative stress, resulting from an imbalance between reactive oxygen species (ROS) production and antioxidant defenses, plays a crucial role in osteoblast differentiation and function [15,16]. NADPH oxidase 2 (NOX2), a key enzyme in reactive oxygen species (ROS) production, is involved in redox signalling, regulating osteoblast differentiation, and bone homeostasis [17,18]. NOX2-derived ROS act as secondary messengers, crucial for osteoblast proliferation and differentiation, but excessive ROS production can lead to cellular damage and impaired bone formation [19]. Superoxide dismutase 2 (SOD2) is an ROS-scavenging enzyme [20], and its deletion impairs osteoblast function [21].

The energy metabolism is tightly linked to osteoblast activity, as these cells demand substantial energy for the synthesis of bone matrix components. Proper mitochondrial function is critical for osteoblast differentiation and mineralisation, while the dysregulation of mitochondrial activity can impair osteoblast function, affecting bone formation [22]. Peroxisome proliferator-activated receptor gamma coactivator 1-alpha (PGC1α) is a transcriptional coactivator that regulates the energy metabolism and mitochondrial biogenesis [23]. It has been reported that PGC-1α enhances the generation of new mitochondria, supporting the energetic demands of osteoblasts and facilitating their differentiation into mature bone-forming cells [24], and the depletion of PGC1α in osteoblasts contributes to decreased bone mass in osteoporosis [25]. Furthermore, mitochondrial transcription factor A (Tfam) is a nuclear-encoded protein primarily known for its role in regulating mitochondrial DNA maintenance [26]. It has been reported that the deletion of Tfam leads to mitochondrial dysfunction of osteoblasts in Tfam-knockout mice and subsequently decreased bone formation [27].

Our aim was to define the ideal culture conditions for MC3T3-E1 preosteoblasts in terms of the mineralising capacity induced by stable chemical formulations for Asc and βGP. For this reason, we selected subclone 4, which has been shown to exhibit a higher calcification rate than other subclones [28]. We also investigated potential pathways that can influence this process and explored if oxidative stress-related molecules and mitochondrial activity can be influenced by different culturing conditions.

## 2. Results

### 2.1. Bone Nodules Are Abundantly Formed under Specific Culture Conditions

To test our hypothesis, MC3T3-E1 subclone 4 preosteoblasts were cultured in osteogenic medium (OSM) using nine different combinations of Asc and βGP concentrations for 21 d to examine their mineralisation capacity. Chemical derivatives for Asc and βGP that are stable and long-acting in cell culture conditions for both factors were used (see Methods). Although a concentration of 50 μg.mL^−^^1^/5 mM Asc/βGP is very effective, based on our previous publications with primary mouse osteoblasts [29,30], a pilot experiment showed no sign of mineralisation with the MC3T3-E1 subclone 4 cell line with the aforementioned concentrations. Therefore, we selected 100, 150, and 200 μg.mL^−1^ of Asc for the subsequent tests and also screened a combination of 2, 3, and 4 mM βGP, as 2 mM is suggested to be very potent in bony structure formation in vitro [14].

**Figure 1:**
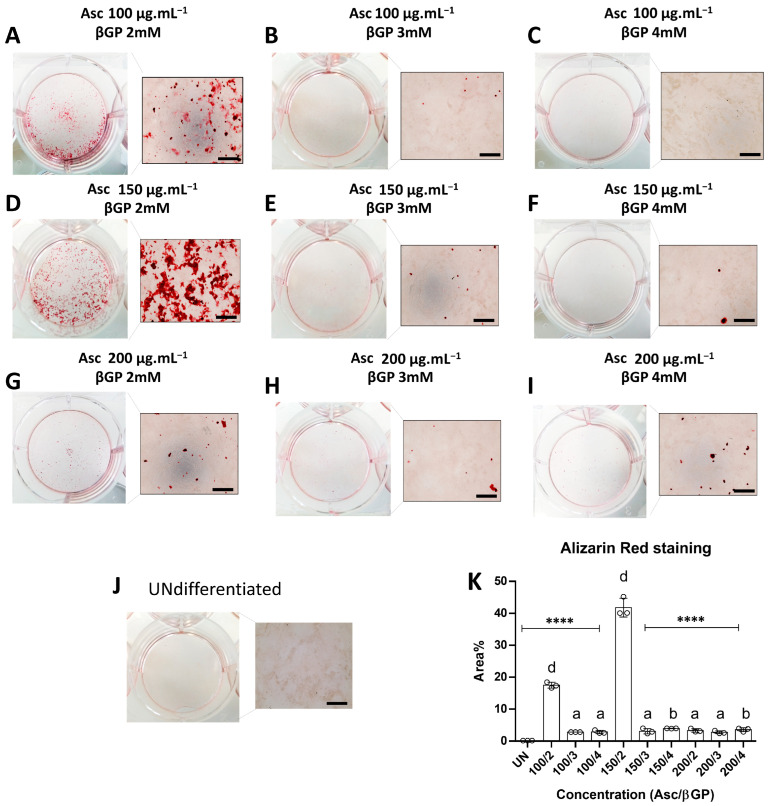
Measurement of calcified area. The effect of different combinations of Asc/βGP in OSM were measured in six-well plates stained by ARS (**A**–**I**) in triplicate (*n* = 3) on day 21 in OSM. Undifferentiated MC3T3-E1 cells (UN) were used as the control (**J**). Quantification of stained areas (**K**) is presented as mean ± SD. **** *p* < 0.0001 compared to 150/2 condition; a for *p* < 0.05, b for *p* < 0.01, and d for *p* < 0.0001, compared to UN; scale bar: 100 μm.

Alizarin Red S (ARS) staining of mineralised bone matrix on day 21 showed a remarkable statistically significant difference between the 150/2 condition and the undifferentiated control, as expected, as well as the other conditions (Figure 1A–J). The 100/2 treatment also demonstrated some limited mineral deposition, but significantly lower compared to 150/2 and higher than in the other conditions (Figure 1K).

### 2.2. Asc/βGP 150/2 Induces Osteogenic Marker Gene Expression and Increased OCN Levels

**Figure 2:**
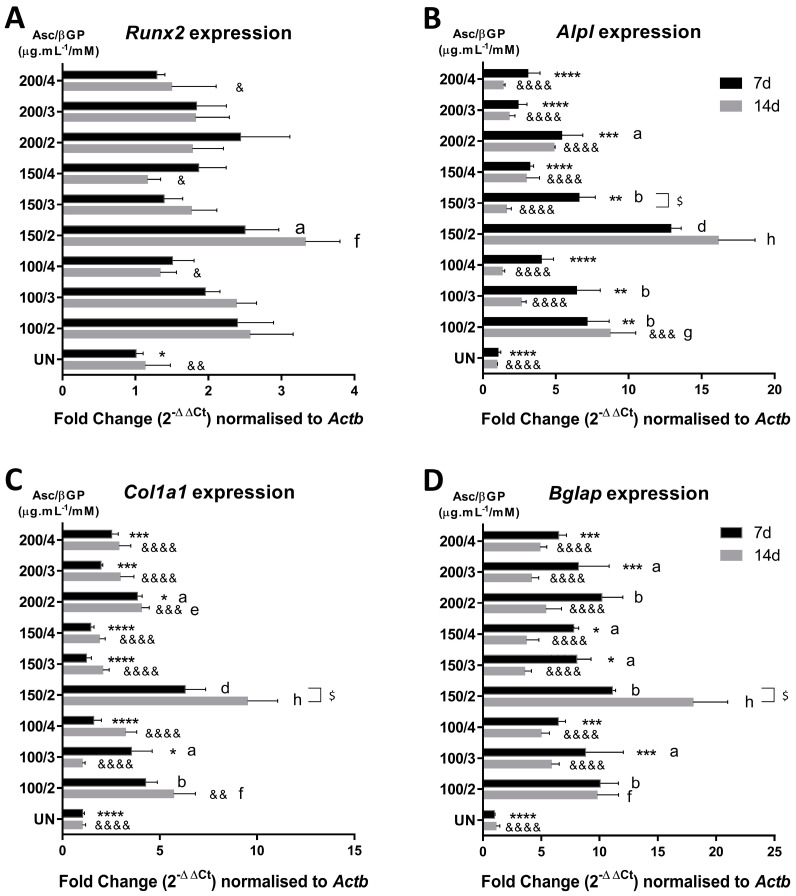
Gene/protein expression of key bone genes on days 7 and 14 in OSM. Runx2, Alpl, Bglap, and Col1a1 gene expression (**A**–**D**) was measured by qPCR and expressed as fold change normalised to Actb. All comparisons were performed on days 7 and 14 by ANOVA, compared to UN and 150/2, as well as between days 7 and 14 for each treatment (*n* = 3). All data are presented as mean ± SD. *, &, a, e for *p* < 0.05; **, &&, b, f for *p* < 0.01; ***, &&&, g for *p* < 0.001; and ****, &&&&, d, h for *p* < 0.0001. Asterisks (*) indicate comparisons with 150/2 on day 7 and ampersands (&) on day 14; letters a, b, and d indicate comparisons versus UN on day 7 and letters e, f, g, and h on day 14; dollars ($ for *p* < 0.05) indicate comparisons between day 7 and day 14 for each condition.

We then explored the early osteoblastic differentiation (7 d) and the progression of bone matrix formation (14 d) events of MC3T3-E1 subclone 4 cells in OSM. Therefore, the gene expression levels of the transcription factor Runx2, a master regulator of osteoblastogenesis, were measured along with Alpl and Bglap, as the mineralisation markers, and Col1a1, as the bone matrix production marker, on days 7 and 14 of culture in OSM. It was found that Runx2 expression was higher for the 150/2 group at both time-points compared to the UN cells. For all the other conditions, an increase was evident but not reaching statistical significance, although the preosteoblastic cells proceeded in a manner similar to osteoblastic differentiation in a variety of Asc/βGP concentrations. In addition, on day 14, Runx2 was significantly lower when 4 mM of βGP was present in the OSM in comparison to 150/2. Alpl, Col1a1, and Bglap gene expression levels were significantly higher in the 150/2 group compared to UN and the other conditions at both time-points (Figure 2B–D). However, for most of the other conditions, a statistical significant increase in these genes versus UN was observed only on day 7 (a, b, and d in Figure 2), while, on day 14, only the genes in groups 100/2 and 150/2 were elevated (e, f, g, and h in Figure 2). It could be also noted that, for the 150/2 condition, there was an increase in gene expression between day 7 and day 14, but only for Col1a1 and Bglap, showing accelerating matrix secretion and bone formation.

**Figure 3:**
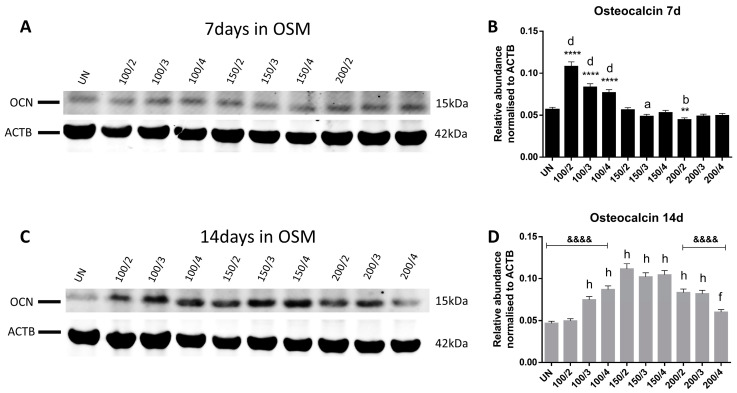
OCN protein abundance. Band intensity in Western blots for OCN on days 7 (**A**) and 14 (**C**) was quantified using the ACTB protein for normalisation and expressed as relative abundance (*n* = 3). OCN protein abundance of all the experimental conditions was compared to 150/2 and UN groups on days 7 (**B**) and 14 (**D**). All data are presented as mean ± SD. **—*p* < 0.01; ****, &&&&—*p* < 0.0001 Asterisks (*) indicate comparisons with 150/2 on day 7 and ampersands (&) on day 14; letters a for *p* < 0.05, b for *p* < 0.01 and d for *p* < 0.0001 indicate comparisons with UN on day 7 and f for *p* < 0.01and h for *p* < 0.0001on day 14.

In addition, Western blot analysis showed that OCN protein levels, which play crucial roles in the mineralisation process, were significantly increased in the 100/2, 100/3, and 100/4 groups compared to the 150/2 on day 7 (Figure 3A,B), but this was reversed at the 14 d time-point, where OCN under the 150/2 treatment was found statistically elevated compared to the other conditions, except for 150/3 and 150/4 (Figure 3C,D), suggesting that, possibly, this Asc concentration induces OCN production. When compared to UN, only 100/2, 100/3, 100/4, 150/3, and 200/4 were found significantly increased on day 7, but all conditions, except for 200/4, presented with significantly elevated OCN levels on day 14.

### 2.3. Culture Medium Composition Controls Bone Matrix Calcification Progress

**Figure 4:**
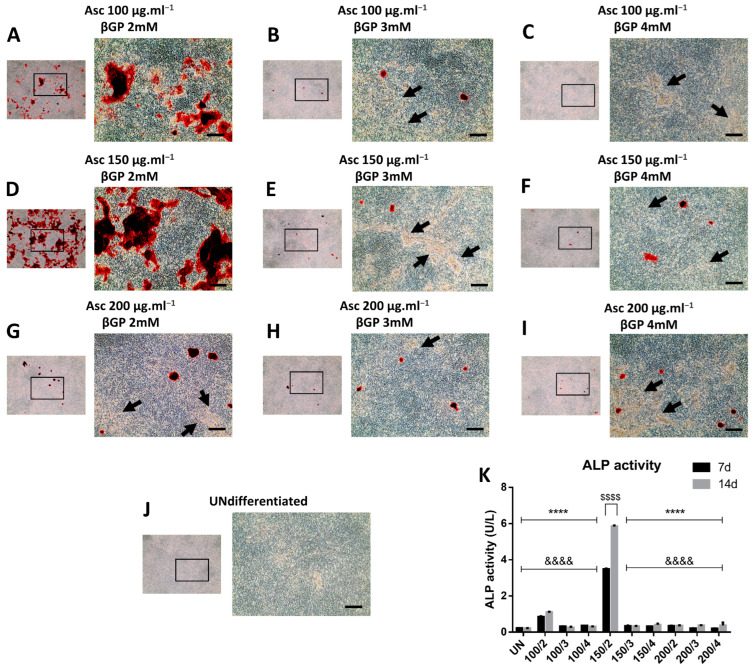
Observation of unmineralised matrix on day 21. Phase-contrast microscopic evaluation of bone matrix production was performed in all the experimental conditions (**A**–**J**). Arrows indicate representative regions where the bone matrix remains unmineralised. ALP activity in the supernatant (*n* = 3) was quantified on days 7 and 14 (**K**) and was expressed as U/L. Data are presented as mean ± SD. ****, &&&&, $$$$ for *p* < 0.0001. Asterisks (*) indicate comparisons with150/2 on day 7 and ampersands (&) on day 14; dollars ($) indicate comparisons between days 7 and 14 for each condition; scale bar: 100 μm.

Furthermore, microscopic imaging revealed that, under all the conditions, the bone matrix was abundantly secreted at the end stage of day 21 (Figure 4A–I), but, as expected, this was less profound in the undifferentiated cells (Figure 4J). A detailed microscopic observation with phase-contrast captions of ARS wells showed that only in 100/2 and 150/2 conditions the bone matrix was properly mineralised and consequently stained (Figure 4A,D), while, in the other conditions, the secreted matrix remained unmineralised or this process was delayed.

Finally, alkaline phosphatase (ALP) activity in the supernatant of the cell cultures on days 7 and 14 clearly indicated that the mineralisation process progressed more rapidly and was more intense in the 150/2 wells, followed by the 100/2 condition, as reflected by the corresponding levels of ALP enzymatic activity (Figure 4K), while, in the other conditions, it seemed that there was a delay in hydroxyapatite crystal formation and deposition. ALP activity was also increased in 150/2 group between days 7 and 14.

### 2.4. Compromised Bone Formation Does Not Implicate Oxidative Stress and Mitochondrial Respiration

Based on our findings, we next aimed to explore whether basic processes, like the energy metabolism and oxidative stress, that regulate the cellular functions of osteoblasts are implicated in the altered osteoblasts’ responses under different experimental conditions.

**Figure 5:**
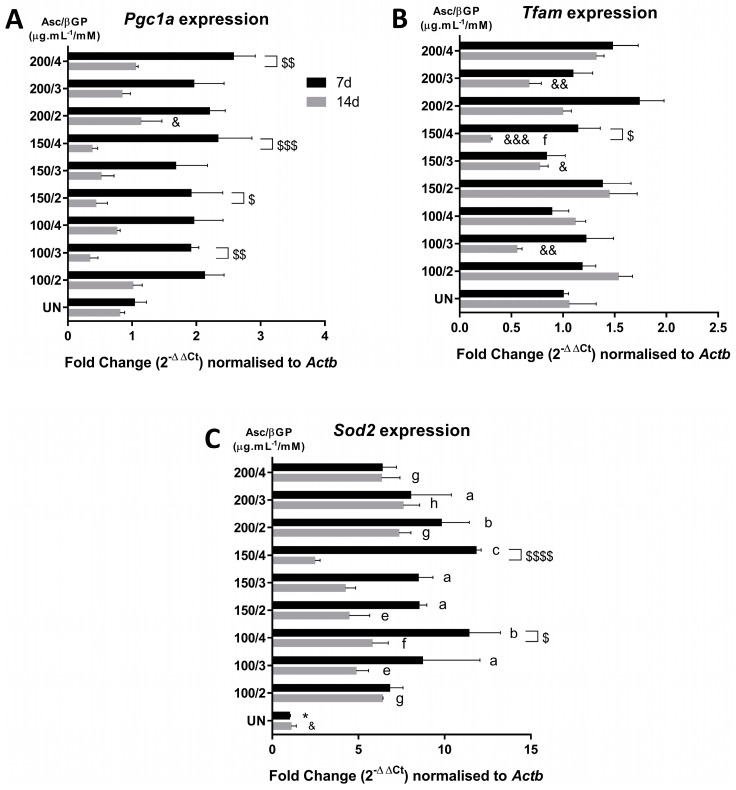
Gene expression of key molecules implicated in oxidative stress and energy metabolism on days 7 and 14 in OSM. Pgc1α, Tfam, and Sod2 gene expression (**A**–**C**) was measured by qPCR and expressed as fold change normalised to Actb. All comparisons were performed on days 7 and 14 by ANOVA (*n* = 3). All data are presented as mean ± SD. *, &, a, e for *p* < 0.05; &&, b, f for *p* < 0.01; &&&, c, g for *p* < 0.001; and h for *p* < 0.0001. Asterisks (*) indicate comparisons with 150/2 on day 7 and ampersands (&) on day 14; letters a, b, and c indicate comparisons with UN on day 7 and letters e, f, g, and h on day 14; dollars ($ for *p* < 0.05; $$ for *p* < 0.01; $$$ for *p* < 0.001; $$$$ for *p* < 0.0001) indicate comparisons between days 7 and 14 for each condition.

We, therefore, measured the gene expression levels of Sod2, which reflects oxidative stress events, and Pgc1α and Tfam, as indicators of mitochondrial biogenesis and the cellular energy metabolism. Although the analysis of Pgc1α on days 7 or 14 for the different culture conditions revealed no differences compared to UN or 150/2 (Figure 5A), all the conditions showed decreased Pgc1a expression from day 7 to day 14, which reached statistical significance in the 100/3, 150/2, 150/4, and 200/4 groups. Suppressed levels of Tfam expression were found in 100/3 and 200/3 as well as in 150/3 and 150/4 compared to 150/2 but not to the UN control (Figure 5B), where no changes were observed between days 7 and 14, except for the 150/4 condition. Finally, the Sod2 expression levels for all the conditions were statistically increased in comparison to UN (Figure 5C). However, no differences were found with the 150/2 condition at both time-points, except for the UN group, and between days 7 and 14 there was a significant decrease only in the 100/4 and 150/4 groups.

**Figure 6:**
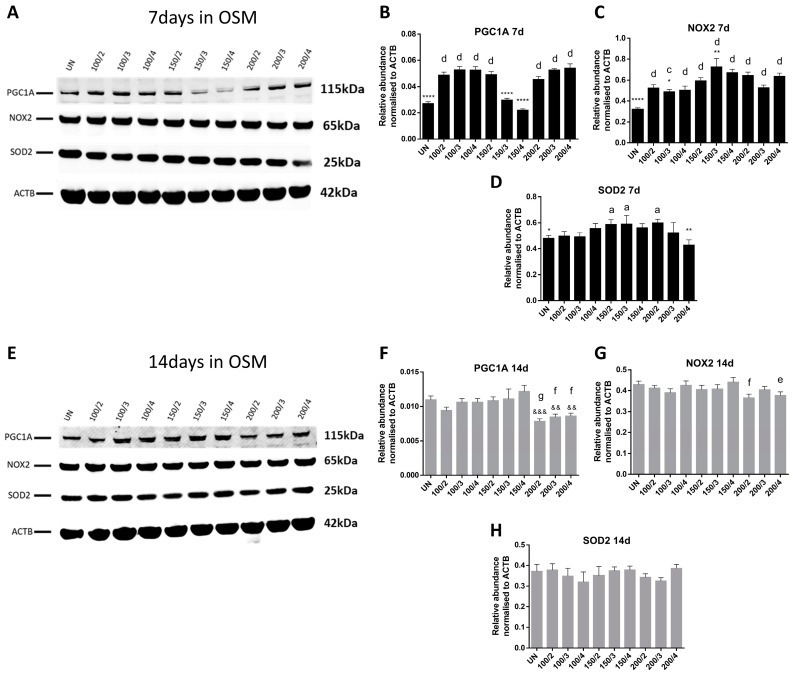
PGC1A, NOX2, and SOD2 protein levels. Band intensity in Western blots for PGC1A, NOX2, and SOD2 on days 7 (**A**–**D**) and 14 (**E**–**H**) was quantified using the ACTB protein for normalisation and expressed as relative abundance compared to the 150/2 and UN groups (*n* = 3). All data are presented as mean ± SD. *, a, e for *p* < 0.05; **, &&, f for *p* < 0.01; &&&, c, g for *p* < 0.001; and ****, d for *p* < 0.0001. Asterisks (*) indicate comparisons with 150/2 on day 7 and ampersands (&) on day 14; letters a, c, and d indicate comparisons with UN on day 7 and letters e, f, and g on day 14.

Similarly, the results obtained from the Western blots for the PGC1A, SOD2, and NOX2 protein levels for days 7 (Figure 6A–D) and 14 (Figure 6E–H) in OSM showed no particular pattern. Statistically significant differences were found in different conditions and time-points, such as for PGC1A for UN, 150/3, and 150/4 conditions compared to 150/2, while in most of the conditions, except for 150/3 and 150/4, the protein levels were elevated compared to UN on day 7 (Figure 6B). At this 7 d time-point, the NOX2 levels were increased in all the conditions in comparison with UN (Figure 6C), while SOD2 were raised only for 150/2, 150/3, and 200/2 (Figure 6D). On day 14, the PGC1A levels were decreased in the 200/2, 200/3, and 200/4 groups compared to both UN and 150/2 (Figure 6F), whereas NOX2 was reduced in 200/2 and 200/4 compared to the UN control (Figure 6G), while no differences were found for SOD2 (Figure 6H).

## 3. Discussion

In vitro cell model systems resemble the physiological events of osteogenesis and calcification, and their use has been useful in the preclinical evaluation of therapeutic agents targeting bone diseases. Among the cells with osteogenic potential, MC3T3-E1 cells are the most established model, and different subclones have been isolated and characterised [31]. However, although these cells are commercially available, with numerous publications using this cell line, there is often a lack of information regarding the use of a specific subclone in the published work or a lack of consistency in the osteoinductive culture media used. Therefore, in this work, we intended to identify the most potent osteogenic culturing conditions for MC3T3-E1 subclone 4 cells, which have been shown to exhibit good osteogenic potential compared to other subclones [12,31]. We report that OSM containing Asc/βGP 150 μg.mL^−1^/2 mM induced cells to produce abundant mineralised bone nodules on day 21, where alternative combinations failed to achieve this and were limited to the secretion of uncalcified bone matrix. In addition, the production of calcified matrix under this condition was accompanied by significant differences in key osteogenic gene markers in comparison with other Asc/βGP combinations. Finally, there was no clear implication of oxidative stress and energy metabolism in this procedure, as revealed by our analysis of the key factors involved in these processes.

Asc and βGP are supplements that are used to induce differentiation in the osteoblastic lineage as well as promote bone-forming capacities [12,31], since spontaneous mineralisation is not observed in in vitro systems [32,33]. Although the function of Asc is well characterised, mainly participating in procollagen synthesis and mature collagen assembly as a crucial cofactor of propyl/lysyl hydroxylases [34,35,36], the exact role of βGP has not been fully uncovered, besides it being a substrate for hydrolysis by alkaline phosphatase. Furthermore, the βGP concentrations used in the literature vary between 2 and 10 mM, depending on the cell type (cell lines, primary, bone marrow mesenchymal stem cells, etc.) [28,37]. Several studies have explored the osteogenic potential of MC3T3-E1 subclone 4 cells as well as primary osteoblastic cells under different conditions. However, it should be noted that the responses of this particular cell line and these primary cells may vary depending on the osteoinductive environment. Furthermore, differences characterise the osteogenic potential between closely related species like mice and rats. For example, mouse primary cells mineralise in αMEM and need up to 28 d to complete mineralisation, while rat primary osteoblasts exhibit mature mineral deposition in both αMEM and DMEM in shorter periods of time (14 d), depending also on the anatomical source of isolation, i.e., calvarial or long-bone-derived osteoblasts (around 20 d for the latter) [6]. In addition, osteogenic supplement concentrations’ effects on the mineralisation of rat and mouse primary osteoblasts differ, where βGP at 2 mM results in abundant trabecular bone-like nodules in rat cell cultures, and higher concentrations (5–10 mM) induce cell death and non-specific ARS staining, while mouse cells can endure up to 5 mM without non-specific staining [9,14]. We used, in our experimental setting, a concentration range of 2–4 mM βGP and found that 2 mM is more efficient in bone nodule formation by MC3T3-E1 subclone 4 cells, consistent with Chung et al., who showed that higher concentrations can lead to the formation of calcium-phosphate crystals other than hydroxyapatite [38]. Therefore, it is crucial to use a low βGP concentration when assessing mineralisation in order to ensure a real effect and avoid unspecific widespread calcification. Furthermore, Asc seems to be utilised in a wide range of concentrations as well, but, in most cases, conventional Asc is added to the OSM at a range of 50–100 μg.mL^−1^ [39,40,41,42]. We sought to explore the effect of the long-lasting L-Ascorbic acid 2-phosphate in our culture system and found that 150 μg.mL^−1^ resulted in the formation of specific and abundant bone nodules. Additionally, it is important to note that we did not add dexamethasone (Dex) in our OSM, as it is known that Dex exposure results in the inhibition of primary murine osteoblasts’ differentiation and formation [43] and MC3T3-E1 cells [44], while it facilitates osteogenic performance in rat cells [45] and promotes osteogenic differentiation in human mesenchymal stem/stromal cells in vitro, where it is an essential additive and is widely used [46,47,48,49].

In the current literature, there is a variety of Asc/βGP combinations used for MC3T3-E1 subclone 4 cells, e.g., 100 μM/2 mM [28], 50 μg.mL^−1^/5 mM [50], 200 μM/10 mM [51], 25 μg.mL^−1^/5 mM [52], and 50 μg.mL^−1^/4 mM [53], while inorganic phosphate is also used in other forms such as NaH_2_PO_4_ [54] or KH_2_PO_4_ [55]. Consequently, cells respond differently in diverse osteogenic conditions, which produces various levels of mineralisation and specificity in ARS staining across publications. Although the most common combination of Asc/βGP is 50 μg.mL^−1^/10 mM in published works using the MC3T3-E1 subclone 4 preosteoblasts, it seems that there is often non-specific ARS staining, probably due to the properties of other formulations of Asc and βGP, the duration of culture in the osteogenic medium, the number of cell passages, or other unexplored reasons. A crucial factor is the passaging of MC3T3-E1 cells, as it seems that a high number of passages results in decreased mineralisation and alters the gene expression of osteoblastic markers [56]. To avoid complications due to this factor, we used a low passaging number, up to 8 for MC3T3-E1 subclone 4 cells, although higher numbers have been used, like between 10 and 20 [57,58], up to 15 [59], or even higher, between 25 and 30 [60].

In addition, we report that this Asc form can induce osteogenic markers’ gene expression in agreement with previous reports [61,62]. However, this was more evident and statistically significant in the 150/2 condition compared to the other experimental conditions and consistent throughout the time course of the experimental period. Similar to our results for ARS staining, Hwang et al. used a combination of 100 μM/2 mM of Asc/βGP and found a good level of osteogenic performance in the same MC3T3-E1 subclone 4, but not in subclones 14 and 24, while *Bglap* gene expression showed a remarkable increase as opposed to *Col1a1*, which was downregulated on day 21 [28]. In our work, *Col1a1* gene expression analysis showed a significant and persistent increase in the 150/2 group at both 7 d and 14 d time-points, forming a mature bone ECM, which was subsequently calcified [63]. Notably, cells in alternative conditions secreted bone matrix as well on day 21 in OSM, but these nodules remained unmineralised. Using a combination of 50 μg.mL^−^^1^/5 mM, Toor et al. found that the peak in *Col1a1*, *Runx2*, and *Alpl* gene expression was on day 7, and a gradual decrease was noted on days 14 and 21; however, calcification was evident, although non-specific ARS staining could be observed [50]. In our experimental settings, *Alpl* and *Bglap* gene levels were elevated in all conditions on day 7 compared to the undifferentiated cells, while a decrease was observed on day 14 in cells treated with 3 mM and 4 mM of βGP, except for 100/2, which remained unchanged and showed a degree of mineralisation, and in 150/2, where ossification was abundant, highlighting that βGP at 2 mM is the most potent osteoinductive condition. In contrast, the OCN protein showed an increase in the 150/2 combination only on day 14, similarly with previous findings [64]. Finally, the *Runx2* levels did not differ among the experimental conditions and were found elevated compared to the undifferentiated cells, although not reaching statistical significance, showing that MC3T3-E1 subclone 4 preosteoblastic cells can be differentiated and form mature osteoblasts at various combinations of Asc/βGP. This finding is similar to previous works, where it has also been reported that MC3T3-E1 cells, without referring to a specific clone, can produce mineralised bone matrix even without osteogenic factors, but this needs further investigation [37]. It is important to note that our ARS staining using the 150/2 combination was highly specific, while only a few studies in the literature showed a similar selectivity, for example, using 2 mM of βGP but a lower Asc concentration [28]; however, in these articles, cells were maintained in OSM for 15–18 d, and, consequently, control MC3T3-E1 subclone 4 cells showed limited mineralisation compared to the other conditions used, which were efficiently stained [65,66]. Collectively, our results indicate that the differences observed between the nine Asc/βGP combinations used were caused by alterations in the mineralisation capacity, as a result of alkaline phosphatase and osteocalcin activity changes, rather than a phenotypic modification, showing that their role is crucial [67,68]. It is also important to note that the reported changes and the delay in biomineralization refer to a specific experimental time course of 21 d in OSM.

In an effort to further explore the cause of the aforementioned results, we evaluated key molecules of oxidative stress and the energy metabolism, cellular processes which affect bone health and bone-related diseases such as osteoporosis [16,69,70,71,72,73,74]. We first examined the gene and/or protein expression levels of Pgc1a and Tfam, important factors of mitochondrial biogenesis in bone cells [24]. It is known that the AMPK/PGC1α axis is activated during osteoblast differentiation in C3H10T1/2, and Tfam is also up-regulated in osteoblast-like MG63 osteosarcoma cells [75,76]. However, we found that *Pgc1a* was reduced on day 14 compared to day 7 in most of the conditions, suggesting a suppression of mitochondrial activity on a transcriptional level, which was not accompanied by the respective protein levels. Similarly, *Tfam* did not show any specific trend. On the other hand, osteoblast-specific Sod2-deficient mice develop an osteoporotic phenotype [21], while Nox2 increase leads to osteocyte apoptosis [77] and glucocorticoid-induced preosteoblast death [78]. Although NOX2 was elevated on day 7 compared to UN, this was reversed on day 14, showing no significant changes between the groups. Furthermore, it has been reported that SOD2 is required for osteoblast differentiation in MC3T3-E1 cells [20], which agrees with our findings on day 7 for 150/2, whereas no differences were observed for SOD2 on day 14, a time-point by which the cells had already undergone differentiation. Finally, we did not find any important patterns between our experimental conditions for these genes/proteins, a result which supports our main conclusion that the mineralisation process is the key difference in the current study.

## 4. Materials and Methods

### 4.1. Cell Culture

The MC3T3-E1 subclone 4 (ATCC CRL-2593) murine preosteoblastic cell line was obtained from the American Tissue Culture Collection (ATCC, Rockville, MD, USA). Alpha-Minimum Essential Medium (α-MEM) supplemented with foetal bovine serum (FBS) is commonly used as a basal medium for culturing MC3T3-E1 cells [5]. Cells (with a low passage number, up to 8) were cultured in alpha-MEM with GlutamaxTM (Gibco, London, UK) and nucleosides, containing 10% heat-inactivated FBS and penicillin (100 IU/mL)/streptomycin (100 μg.mL^−1^) (Invitrogen, Paisley, UK), in a humidified 5% CO_2_ incubator at 37 °C, as previously described [29,30]. Upon reaching confluence, the cells were harvested using trypsin/EDTA (Gibco, London, UK) and seeded onto 6-well plates (10^5^ cells/well). All the wells contained cells from the second passage. Two days after seeding and the adherence of the cells, 2 mL of OSM containing different concentrations of Asc and βGP (Sigma, Poole, UK) was added to the wells. The actual form for these OSM factors was the following: Asc, L-Ascorbic acid 2-phosphate sesquimagnesium salt hydrate (Sigma, Poole, UK, A8960); and βGP, β-Glycerophosphate disodium salt hydrate (Sigma, Poole, UK, G9422). The combinations of Asc/βGP concentrations were the following (μg.mL^−1^ Asc/mM βGP): 100/2, 100/3, 100/4, 150/2. 150/3, 150/4, 200/2, 200/3, and 200/4 (9 experimental conditions). Cells without OSM (undifferentiated, UN) served as the control. All the conditions were assessed in triplicate, and, in all the wells, the conditioned medium was changed every 3 days with freshly prepared OSM. Three sets of time-points were used for this analysis: 7 d and 14 d for gene expression by qPCR, ALP activity by spectrophotometry, and protein abundance by Western blot; and 21 d for terminal mineralisation evaluation.

### 4.2. Bone Nodule Staining

Mineralisation capacity was assessed by ARS (Sigma, Poole, UK) staining. In brief, on the 21st day of culture in OSM, the wells were washed twice with PBS (without Ca^2+^), and the cells were fixed with 1 mL of 10% neutral buffered formalin (NBF) for 20 min. Following three additional washes with PBS, 1 mL of 4 mM ARS (pH 4.2) was added for 30 min, and the wells were extensively washed with distilled H_2_O until no colour could be observed in the washes. The wells were photographed, and 10× pictures were taken with a Leica DM IL LED (Leica, London, UK) inverted brightfield microscope. The bone nodule surface area was calculated using ImageJ v2.1.4.7 (NIH), as previously described [29,30].

### 4.3. Alkaline Phosphatase (ALP) Activity Assay

The ALP activity assay in the cell culture supernatants was conducted following a 7 d and 14 d period of culture with OSM. Twenty-four hours before supernatant collection, the culture medium was replaced αMEM without phenol (Gibco, London, UK) OSM to avoid interference with the colorimetric method. Using 96-well plates, equal volumes (50 μL) of p-nitrophenyl phosphate (p-NPP) in a 0.01 M glycine buffer (pH 10.5) solution were added to 100 μL of supernatants (in duplicate), followed by incubation for 60 min at 37 °C. The reaction was terminated by adding 20 μL of 1 N NaOH. The enzyme activity of p-NPP hydrolysis was measured with a microplate reader at 405 nm, and p-nitrophenol (p-NP) was used to construct the standard curve. ALP activity was expressed as Units/L.

### 4.4. Quantitative Polymerase Chain Reaction (qPCR)

To compare gene expression levels, total RNA was isolated from MC3T3-E1 cells of all conditions from 3 biological replicates on days 7 and 14 of culture using TRiZOL reagent (Invitrogen, Paisley, UK) and cleaned-up using the RNeasy kit (Qiagen, London, UK), and cDNA was synthesised with the High Capacity cDNA Transcription kit (Applied Biosystems, Warrington, UK) using 1 μg of purified RNA. RNA quality and quantity were evaluated by the 260/280 nm ratio using a NanoDropTM 2000 device (Thermo Scientific, London, UK). Expression levels were measured for alkaline phosphatase (*Alpl*), collagen type 1 (*Col1a1*), runt-related transcription factor 2 (*Runx2*), bone gamma-carboxyglutamic acid-containing protein (*Bglap*), peroxisome proliferator-activated receptor gamma coactivator 1-alpha (*Pgc1α*), transcription Factor A mitochondrial (*Tfam*), and superoxide dismutase (*Sod2*) using specific primers from our previous works [29,79] (Table S1). Real-time qPCR was performed in 20 μL reaction volumes using a RotorGene 6000 (Corbett Research) instrument with SYBR (Bioline, London, UK), and the results were analysed using beta-actin (*Actb*) as a stable reference gene for murine osteoblasts. The qPCR conditions were as follows: 95 °C for 10 s, 60 °C for 15 s, 72 °C for 30 s (35 cycles), using a hot start step of 95 °C for 15 s. The results were analysed using the delta delta CT method (2^−ΔΔCt^) and presented as fold change compared to the reference gene expression levels [80].

### 4.5. Western Blotting

Along with RNA isolation, total protein was extracted from the MC3T3-E1 cells after 7 d and 14 d in OSM under all the experimental conditions. The cells were lysed with RIPA Lysis Buffer (Sigma, Poole, UK) containing Pierce Protease Inhibitors (ThermoFisher, London, UK), and the samples were homogenised by sonication for 30 s, twice, and then centrifuged for 10 min at 14,000× *g* at 4 °C. The supernatant was collected, and the total protein content was determined with a BCA Protein Assay (Sigma, Poole, UK). A total of 20 μg protein extract for each sample was separated by electrophoresis on NuPAGE™ 4–12%, Bis-Tris Mini Protein Gel (Invitrogen, Renfrewshire, UK), and transferred to a polyvinylidene fluoride (PVDF) membrane. Actin β (ACTB) was used as the loading control. The membranes were blocked with 5% bovine serum albumin (BSA) (Sigma, Poole, UK) in tris-buffered saline (TBS) for 1 h and probed with primary antibodies overnight at 4 °C. After incubating with a fluorescence secondary antibody (Licor rabbit anti-mouse, 1:10,000), the blots were imaged on Licor Odyssey CLx (Licor, Bad Homburg, Germany), and the band densities were analysed using ImageJ v92.1.4.7. Band quantifications were normalised to the reference protein.

The primary antibodies are as follows: anti-NOX2/gp91phox (ab129068, dilution 1:400), anti-ACTB (ab8227, 1:2000), and anti-OCN (ab93876, 1:500) from Abcam (Cambridge, UK); anti-SOD2 (ADI-SOD-111, 1:3000) from Enzo Life Sciences (Farmingdale, New York, NY, USA); and anti-PGC1α (NBP1-04676, 1:400) from Novus Biologicals (Abingdon, UK).

### 4.6. Statistical Analysis

All data were analysed with the GraphPad Prism 9 software and expressed as the mean ± SD. The data sets were tested for Gaussian distribution with the D’Agostino–Pearson normality test. Comparisons between the experimental conditions were performed by a one-way analysis of variance (ANOVA), followed by Dunnett’s multiple comparisons post hoc test. Comparisons between days 7 and 14 for each group were performed with the Šidák correction. In all cases, *p* values less than 0.05 were considered statistically significant.

## 5. Conclusions

In conclusion, here we defined the most potent osteogenic culture conditions in which MC3T3-E1 subclone 4 preosteoblastic cells differentiate into mature osteoblasts and produce a bone matrix that is mineralised, resulting in a sufficient number of bone nodules quantifiable by ARS staining on day 21. This OSM is specifically supplemented with 150 μg.mL^−1^ of L-Ascorbic acid 2-phosphate sesquimagnesium salt hydrate and 2 mM of β-Glycerophosphate disodium salt hydrate. In addition, this finding is supported by a significant increase in osteogenic markers and ALP and OCN proteins, which are key regulators of calcification. In combination with the gene/protein expression results of Pgc1a, Tfam, Sod2, and Nox2, we conclude that the final outcome in bone formation is due to the effect of the specific OSM on mineralization, with no implication of oxidative stress or mitochondrial biogenesis processes. Our results could be used as a standard protocol from other research teams and could assist in obtaining reliable and repeatable results in the preclinical testing of osteoanabolic agents without under- or overestimation of the mineralisation outcome.

## Data Availability

All data is available from the corresponding author upon reasonable request.

## Supplementary Materials

The following supporting information can be downloaded at https://www.mdpi.com/article/10.3390/ijms25084180/s1.
