# Supplementary material for: Defining the Most Potent Osteoinductive Culture Conditions for MC3T3-E1 Cells Reveals No Implication of Oxidative Stress or Energy Metabolism
Source: Int J Mol Sci. 2024 Apr 10;25(8):4180. doi: 10.3390/ijms25084180 (PMC11050066; doi:10.3390/ijms25084180)
Supplement: Supplementary file 1 [file ijms-25-04180-s001.zip › ijms-2918940-supplementary.pdf]

Table S1: Sequences of the primers used for qPCR.

Gene	Primer sequences
Alpl	Forward: 5'-CCTTGAAAAATGCCCTGAAA-3'<br>Reverse: 5'-CTTGGAGAGAGCCACAAAGG-3'
Col1a1	Forward: 5'-TGGCCCCATTGGTAACGTTGGT-3'<br>Reverse: 5'-AGGACCTTGTTTGCCGGGTTCA-3'
Runx2	Forward: 5'-TCTGCCGAGCTACGAAATGCCT-3'<br>Reverse: 5'-TGAAACTCTTGCCTCGTCCGCT-3'
Bglap	Forward: 5'-CATGAGGACCCTCTCTCTGC-3'<br>Reverse: 5'-TGGACATGAAGGCTTTGTCA-3'
Pgc1α	Forward: 5'-GACAGGTGCCTTCAGTTCAC-3'<br>Reverse: 5'-CAACCAGAGCAGCACACTCTA-3'
Sod2	Forward: 5'-TTAACGCGCAGATCATGCA-3'<br>Reverse: 5'-GGTGGCGTTGAGATTGTTCA-3'
Tfam	Forward: 5'-CATAGGCACCGTATTGCGTG-3'<br>Reverse: 5'-TCGGAATACAGACAAGACTGATAGA-3'
Actb	Forward: 5'-GCCCTGAGGCTCTTTTCCAG-3'<br>Reverse: 5'-TGCCACAGGATTCCATACCC-3'
